# Cerebral Venous Sinus Thrombosis following COVID-19 Vaccination: Analysis of 552 Worldwide Cases

**DOI:** 10.3390/vaccines10020232

**Published:** 2022-02-03

**Authors:** Cesare de Gregorio, Luigi Colarusso, Giuseppe Calcaterra, Pier Paolo Bassareo, Antonio Ieni, Anna Teresa Mazzeo, Giuseppe Ferrazzo, Alberto Noto, Ioanna Koniari, Jawahar L. Mehta, Nicholas G. Kounis

**Affiliations:** 1 Department of Clinical and Experimental Medicine, Division of Cardiology, G. Martino University Hospital Medical School of Messina, 98125 Messina, Italy; luigi.colarusso@alice.it (L.C.); fergiu89@libero.it (G.F.); 2 Department of Cardiology, Postgraduate Medical School of Cardiology, University of Palermo, 90127 Palermo, Italy; peppinocal7@gmail.com; 3 Department of Cardiology, Mater Misericordiae University Hospital Crumlin, University College of Dublin, D07R2WY Dublin, Ireland; piercard@inwind.it; 4 Pathology Unit, Department of Human Pathology G. Barresi, G. Martino University Hospital Medical School of Messina, 98125 Messina, Italy; antonio.ieni@unime.it; 5 Department of Human Pathology G. Barresi, Division of Anesthesia and Critical Care, G. Martino University Hospital Medical School of Messina, 98125 Messina, Italy; annateresamazzeo@unime.it (A.T.M.); alberto.noto@unime.it (A.N.); 6 Department of Cardiology, University Hospital of South Manchester, NHS Foundation Trust, Manchester M23 9LT, UK; iokoniari@yahoo.gr; 7 Department of Medicine, University of Arkansas for Medical Sciences and the Veterans Affairs Medical Center, Little Rock, AR 72205, USA; mehtajl@uams.edu; 8 Department of Internal Medicine, Division of Cardiology, University of Patras Medical School, 26221 Patras, Greece; ngkounis@otenet.gr

**Keywords:** cardiovascular disease, cerebral venous sinus thrombosis, COVID-19 pandemic, side effects, vaccination

## Abstract

To date, billions of vaccine doses have been administered to restrain the current COVID-19 pandemic worldwide. Rare side effects, including intravascular blood clots, were reported in the general population after vaccination. Among these, cerebral venous sinus thrombosis (CVST) has been considered the most serious one. To shed further light on such an event, we conducted a literature search for case descriptions of CVST in vaccinated people. Findings were analyzed with emphasis on demographic characteristics, type of vaccine, site of thrombosis, clinical and histopathological findings. From 258 potential articles published till September 2021, 41 studies were retrieved for a total of 552 patients. Of these, 492 patients (89.1%) had received AZD1222/Vaxzevria, 45 (8.2%) BNT162b2/CX-024414 Spikevax, 15 (2.7%) JNJ-78436735, and 2 (0.3%) Covishield vaccine. CVST occurred in 382 women and 170 men (mean aged 44 years), and the median timing from the shot was 9 days (range 2–45). Thrombi were predominantly seen in transverse (84%), sigmoid (66%), and/or superior sagittal (56%) sinuses. Brain injury (chiefly intracranial bleeding) occurred in 32% of cases. Of 426 patients with detailed clinical course, 63% were discharged in good clinical conditions, at times with variable neurological sequelae, whereas 37% deceased, largely due to brain injury. This narrative review confirmed CVST as a rare event after (adenoviral vector) COVID-19 vaccination, with a women/men rate ratio of 2.25. Though the pathogenesis of thrombosis is still under discussion, currently available histopathological findings likely indicate an underlying immune vasculitis.

## 1. Introduction

A collective immunization is an important step forward in weathering the COVID-19 pandemic. However, side effects have been reported after vaccine shots. Rare serious adverse events such as vascular syndromes, myocarditis, pericarditis, neurological disease, and other illnesses have been described in a variable proportion of recipients [1,2,3,4,5]. Vascular thrombosis occurred in some individuals, particularly young women, at times surprisingly involving the cerebral venous sinus (CVS) vessels. Blood clots were often associated with thrombocytopenia, and a novel vaccine-induced immune thrombotic thrombocytopenia (VITT) syndrome was established as of February 2021 [5,6]. Since then, a rising body of literature on such cases and possible pathophysiological mechanisms have been published. The VITT syndrome closely resembles the heparin-induced thrombocytopenia (HIT) identified first by Kelton in 1986 [7] and more recently reprised by Greinacher [8], with the difference that VITT is not triggered by heparin administration.

In addition, in the general population, CVS thrombosis (CVST) has been considered a very rare disease, affecting as many as one in 1,000,000 people and one in 5000 to 15,000 hospitalized patients for various diseases [9,10].

As early cases of CVST after vaccination appeared in the literature [11], a warning on neurological disorders occurring within 15 days after the first shot was provided by health authorities all over the world. In March 2021, the European Medicines Agency reported a few cases of CVST thrombosis related to vaccines [12], but many more were described afterwards.

In order to shed further light on the main clinical, pathophysiological, and prognostic features of this new syndrome, we conducted a literature search of published reports describing CVST after COVID-19 vaccination.

## 2. Materials and Methods

A literature search through PubMed, Medline, Life science journals, and SCOPUS database was performed for case series and single reports of CVST following COVID-19 vaccination, published between December 2020 and September 2021. The following MESH terms were used for searching: “acute cardiovascular events”, “cardiovascular disease”, “COVID-19”, “cerebral vein/venous thrombosis”, “deep venous thrombosis”, “SARS-CoV-2”, “side-effects”, “thrombosis”, “vaccine”, together with “ChAdOx”, “AstraZeneca”, “Vaxzevria”, “Ad26.COV2.S”, “Janssen”, “Johnson”, “mRNA-1273”, “Moderna”, “BNT162b2”, “Pfizer”, “Comirnaty”, were applied. We first evaluated the titles and abstracts of each article to be suitable for inclusion in this review. Studies must have been written in English, reporting CVST as by cranial computed tomography or magnetic resonance imaging after vaccine shots, in the context of a VITT syndrome. Clinical features, type of vaccine, demographic characteristics, imaging, sites of CVST, prevalence of thrombocytopenia and PF4-polyanion antibodies, therapeutic approaches, and histopathological findings, were anonymously recognized and analyzed.

## 3. Results

### 3.1. Patient Population

**Figure 1:**
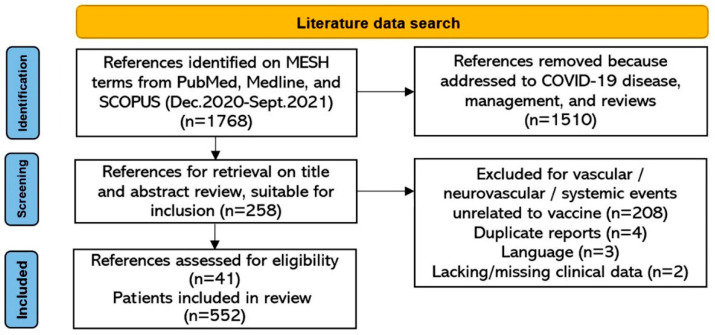
Flow diagram for study selection.

From a total of 258 potential articles, after excluding COVID-related CVST, potential duplicates, multiple reports of the same dataset, redundant and incomplete reports, 41 eligible studies on 552 patients [5,11,13,14,15,16,17,18,19,20,21,22,23,24,25,26,27,28,29,30,31,32,33,34,35,36,37,38,39,40,41,42,43,44,45,46,47,48,49,50,51] were retrieved (Figure 1).

Three were systematic reviews or case series, consisting of 213 patients from the European Medicines Agency [34] and 197 from the UK [38,39]. Another multicenter registry from Germany also described CVST as a manifestation of the HIT-like VITT syndrome [42]. Basic characteristics of the patient population appear in Table 1.

Although CVST occurred after all type of vaccines, it was much more frequent after ChAdOx1 nCoV-19 shots. patients were free of chronic cardiovascular disease, conventional risk factors, or predisposing pro-thrombotic genetic conditions, often were younger than 50 years of age.

Conversely, thrombophilia mutation(s), antiphospholipid antibodies, C-protein/S-protein deficiency, Leiden factor, were reported in mRNA vaccine recipients, corresponding to 10% of the whole study population.

Some CVST patients presented twice in the emergency department after the first shot, because of atypical or misinterpreted symptoms, especially from January to March 2021, when VITT syndrome had not yet been recognized. However, patients experienced a complex clinical picture, in most cases requiring a multispecialty problem-solving team, with neurologists, neurosurgeons, vascular surgeons, and intensivists.

### 3.2. Demographic and Clinical Characteristics

**Figure 2:**
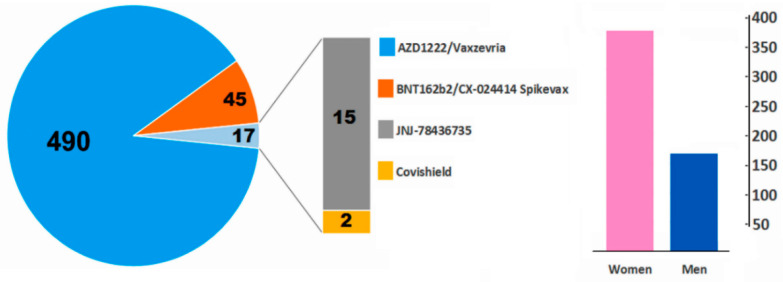
Summary of included cases (*n* = 552) with CVST after vaccination. The women/men ratio was 2.25.

Cerebral venous sinus thrombosis occurred in 382 women and 170 men (W/M ratio 2.25), with a median age of 44 years (range 18–84) (Figure 2). The median time of symptom onset from vaccination (first shot in 99% of cases) was 9 days (range 2–42).

Unrelenting headache, nausea, vomiting, dizziness, seizure, were frequently complained by alert and oriented cases on arrival to the hospital. Some patients, however, presented in the emergency department with a severe neurological syndrome such as hemorrhagic stroke, cerebral edema/infarction, hematomas (hereinafter all these termed brain injury), often in a comatose setting.

### 3.3. Site of Thrombosis

**Figure 3:**
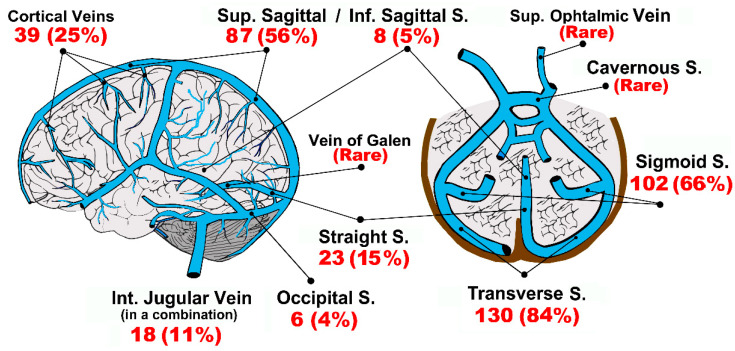
Representation of the anatomy of cerebral veins, site(s) and rates of thrombus formation (*n* = 155 patients). Most patients were found to have clots in more than a single vessel. Abbreviations, S.: Sinus/sinuses; Inf.: Inferior; Int.: Internal; Sup.: Superior.

Thirty-seven studies (*n* = 155 patients) clearly described the location of blood clots within the cerebral circulation. Thrombi were detected in almost all the main venous channels (Figure 3), but predominantly transverse and sigmoid sinuses, with a left-side predominance (53% vs. 47% of right-side, *p* = NS). Five percent of patients showed bilateral transverse, sigmoid, or cerebral vein thrombosis.

**Table 1:** Patient population from 41 studies from all over the world.

Author	Females	Males	Age *	Country	Vaccine
(1) Esba et al. [15]	1	1	50	Saudi Arabia	AZD1222 (ChAdOx1 nCoV-19)
(2) Aladdin et al. [16]	1	−	36	Saudi Arabia	AZD1222 (ChAdOx1 nCoV-19)
(3) Bayas et al. [17]	1	−	55	Germany	AZD1222 (ChAdOx1 nCoV-19)
(4) Bjørnstad-Tuveng et al. [18]	1	−	30	Norway	AZD1222 (ChAdOx1 nCoV-19)
(5) Choi et al. [19]	−	1	33	Korea	AZD1222 (ChAdOx1 nCoV-19)
(6) Crossette-Thambiah et al. [20]	4	−	44	UK	AZD1222 (ChAdOx1 nCoV-19)
(7) D’Agostino et al. [11]	1	−	54	Italy	AZD1222 (ChAdOx1 nCoV-19)
(8) De Michele et al. [21]	2	−	56	Italy	AZD1222 (ChAdOx1 nCoV-19)
(9) Dias et al. [22]	2	−	57	Portugal	BNT162b2
(10) Dutta et al. [23]	−	1	51	India	Covishield
(11) Fanni et al. [24]	1	−	58	Italy	AZD1222 (ChAdOx1 nCoV-19)
(12) Franchini et al. [25]	−	1	50	Italy	AZD1222 (ChAdOx1 nCoV-19)
(13) Gattringer et al. [26]	1	1	33	Austria	AZD1222 (ChAdOx1 nCoV-19)
(14) Geeraerts et al. [27]	2	−	N.A.	France	AZD1222 (ChAdOx1 nCoV-19)
(15) George et al. [28]	1	−	40	Colorado (US)	JNJ-78436735 (Ad26.COV2)
(16) Gessler et al. [29]	3	−	47	Germany	AZD1222 (ChAdOx1 nCoV-19)
(17) Graf et al. [30]	−	1	29	Germany	AZD1222 (ChAdOx1 nCoV-19)
(18) Greinacher et al. [5]	9	1	36	Germany/Austria	AZD1222 (ChAdOx1 nCoV-19)
(19) Ikenberg et al. [31]	1	−	30	Germany	AZD1222 (ChAdOx1 nCoV-19)
(20) Jamme et al. [32]	1	−	69	France	AZD1222 (ChAdOx1 nCoV-19)
(21) Kotal et al. [33]	1	−	32	India	Covishield
(22) Krzywicka et al. [34]	139	48	46	EMA survey	AZD1222 (ChAdOx1 nCoV-19)
(22) Krzywicka et al. [34]	20	6	56	EMA survey	BNT162b2/CX-024414 Spikevax
(23) Lin et al. [35]	−	1	52	Taiwan	AZD1222 (ChAdOx1 nCoV-19)
(24) Mazzeo et al. [36]	1	1	50	Italy	AZD1222 (ChAdOx1 nCoV-19)
(25) Mehta et al. [13]	−	2	28	United Kingdom	Vaxzevria
(26) Muir et al. [37]	1	−	48	Nebraska (US)	JNJ-78436735 (Ad26.COV2)
(27) Pavord et al. [38]	57	45	48	UK (MC)	AZD1222 (ChAdOx1 nCoV-19)
(28) Perry et al. [39]	54	41	53	UK (MC)	AZD1222 (ChAdOx1 nCoV-19)
(29) Pomara et al. [40]	1	−	39	Italy	AZD1222 (ChAdOx1 nCoV-19)
(30) Schultz et al. [41]	4	1	39	Norway	AZD1222 (ChAdOx1 nCoV-19)
(31) Schulz et al. [42]	29	8	45	Germany (MC)	AZD1222 (ChAdOx1 nCoV-19)
(31) Schulz et al. [42]	6	2	45	Germany (MC)	BNT162b2
(32) See et al. [14]	12	−	46	VAERS (US)	JNJ-78436735 (Ad26.COV2)
(33) Smadja et al. [43]	3	4	34	France	AZD1222 (ChAdOx1 nCoV-19)
(33) Smadja et al. [43]	4	−−	34	France	BNT162b2
(33) Smadja et al. [43]	3	−	33	France	CX-024414 Spikevax
(34) Syed et al. [45]	−	1	45	Pennsylvania (US)	CX-024414 Spikevax
(35) Suresh et al. [46]	1	−	29	UK	AZD1222 (ChAdOx1 nCoV-19)
(36) Tølbøll Sørensen et al. [44]	1	−	30	Denmark	AZD1222 (ChAdOx1 nCoV-19)
(37) Vayne et al. [47]	5	1	44	France (MC)	AZD1222 (ChAdOx1 nCoV-19)
(38) Wiedmann et al. [48]	5	−	39	Norway	AZD1222 (ChAdOx1 nCoV-19)
(39) Wolf et al. [49]	3	−	36	Germany	AZD1222 (ChAdOx1 nCoV-19)
(40) Yahyavi-Firouz-Abadi et al. [50]	1	−	30	Maryland (US)	JNJ-78436735 (Ad26.COV2)
(41) Zakaria et al. [51]	−	1	49	Malaysia	BNT162b2

* Median value (from case series) or single patient’s age (years). Abbreviations, E.M.A.: European Medicines Agency; M.C.: Multicenter Study/Registry; N.A.: not available; U.K.: United Kingdom; U.S.: United States (of America); V.A.E.R.S.: Vaccine Adverse Event Reporting System.

Internal jugular veins thrombosis was detected in 18 patients, very often in a combination with homolateral transverse and sigmoid sinuses. However, carotid/jugular ultrasound was performed only in a small number of patients.

Multisite systemic venous thromboses were observed in 141/155 patients (91%), mainly involving abdominal districts (mesenteric, hepatic and splanchnic), pulmonary arteries (thrombo-embolism), and inferior limbs. Two patients from De Michele et al.’s publication [21] were recognized to have a combination of cerebral artery and vein occlusion. As shown in Table 2, 32/41 studies met the criteria for VITT [5,8,41,49,52] only in a proportion of patients.

### 3.4. Histopathological Findings

Only scant information was available on histopathological findings in CVST patients. Postmortem examination was performed in the study of Wiedmann et al. [48]. Aside from the description of hemorrhagic areas, one patient was found to have mixed red-white clot formation in the transverse and sigmoid sinus, as well as remnants of white clots attached to the endothelium in the sagittal sinus. Microscopy confirmed multiple arteriolar thrombi in organization. Fibrin aggregates were also observed within the small veins of lung lobes and of the myocardium as well. Another patient showed extracerebral thrombi were rich in platelets, fibrin, leukocytes, and abundant monocytes attached to the endothelium, but disorganized. Of note, those patients had no pre-existing organ disease.

Fibrin thrombi in the cortical vessels and microthrombi in the capillaries of choroid plexuses were reported by Fanni et al. [24]. Those findings were associated with intestinal wall hemorrhagic necrosis, venous congestion in various organs. Micro-thrombi were also seen in the lumen of dilated pulmonary capillary vessels associated with edema, cellular debris, as in the early exudative phase of acute respiratory distress syndrome.

Multivessel thrombi were also seen within the cardiac veins and capillaries, both in the subepicardial fat and deep myocardium, as well as in the vasa vasorum of the arterial wall of the abdominal aorta.

**Figure 4:**
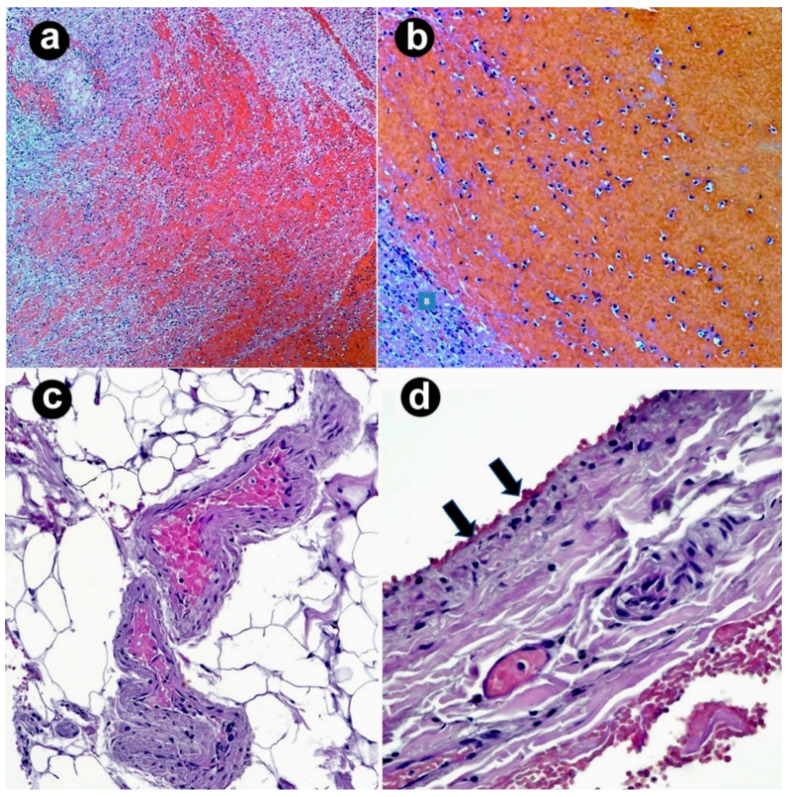
Histopathological findings in a female patient with CVST (hematoxylin-eosin). (**a**) Well-organized thrombus with centrally located red blood cells/hemorrhagic areas and surrounding fibrin accumulations (100× original magnification); (**b**) leukocytes are variably interspersed (200× original magnification); (**c**) microthrombi in small vessels at the periphery of the large thrombus (400× original magnification); (**d**) endotheliitis with mononuclear infiltration and areas of endothelial desquamation (arrows) (400× original magnification).

Figure 4 illustrates a well-organized thrombus with centrally located red blood cells in hemorrhagic areas and surrounding fibrin accumulation in a Sicilian patient with CVST. Microthrombi were also found within the small vessels at the periphery of a large thrombus formation. Interspersed leukocytes and mononuclear infiltration were also present, with large endothelial desquamation.

Tissue inspection by Pomara et al. [40] revealed the presence of thrombi in the small-medium-sized vessels and increased inflammatory infiltrates. Immunohistochemical analyses highlighted the vascular and peri-vascular expression of adhesion molecules such as VCAM-1, as well as the presence of CD66b+, CD163+, and CD61+-activated inflammatory cells (especially leukocytes, both polymorphonuclear cells and monocytes/macrophages and megakaryocytes), suggesting the activation of the innate immune system. Multiple organ injury was also due to the deposition of pro-inflammatory molecules (C1r, C4d) in the interstitial spaces.

### 3.5. Management and Outcomes

Analysis of in-hospital management was not the aim of the present analysis; notably other studies have addressed this aspect [14,34,38,42,55,56,57]. Herein, we summarize the main treatment provided to patients. Intravenous immunoglobulins (Igs) were first administered to most patients at a mean dose of 1 g/kg [5,10,14,34]. Anticoagulants (*argatroban, fondaparinux*, etc.) were also given, except for patients with intracranial bleeding. In general, heparin use was discouraged because of the potential of bursting the PF4-polyanion immunogenicity, according to a shared pathomechanism between HIT and VITT [5,10]. Platelet transfusion was discouraged based on based on lack of information on benefit in patients with HIT [7,8] or disseminated intravascular coagulation (DIC) [58].

Overall, positive outcomes were reported in no-stroke patients. Advanced endovascular procedures such as rheolysis, flushing, and aspiration with 50% saline solution with contrast medium [49] or mechanical thrombectomy [19] were attempted throughout the jugular vein.

As suggested by the current European Stroke guidelines [57], decompressive neurosurgery was performed in patients with parenchymal lesions, edema, and impending herniation. Brain injury was observed in 174 patients (32%).

**Figure 5:**
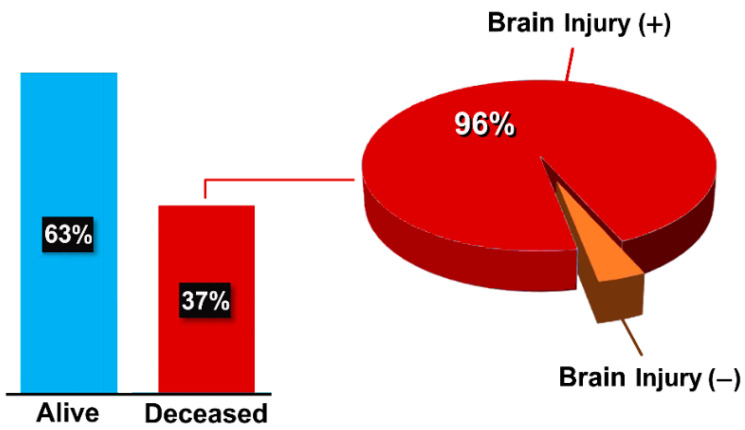
Clinical outcome in the patient population (*n* = 426). Brain injury was described as cerebral edema, hematoma, hemorrhage, and/or infarction, often associated with thrombocytopenia.

However, the clinical course was comprehensively described in 426/552 patients (77%). Two hundred and sixty-eight in this subgroup (63%) were discharged from the hospital in a good clinical condition, at times with variable neurological sequelae, whereas 158 died (37%); 152 of these deaths were in the ChAdOx1nCoV-19 group, and were related to stroke injury and/or thrombocytopenia (Figure 5).

## 4. Discussion

To the best of our knowledge, this is the largest case series analysis of CVST following COVID-19 vaccination since December 2020, the first COVID-19 vaccines received emergency use authorization in the Western countries, until September 2021. Although very dramatic in some patents, CVST was confirmed as a rare clinical event, considering the high number of doses of vaccine administered worldwide.

In general, CVST has been described as a form of vascular disease occasionally affecting young women with the same risk factors as in other forms of peripheral venous thrombosis [57,58,59,60]. As of early April 2021, online published index-cases were from Europe and UK [5,11,13,25,42], but many other patients were reported afterwards.

It was difficult to provide the true incidence of CVST in a real-world vaccinated population, also because suspected, probably, or not recognized cases may have never been published. Studies on the US general population established a rate of 0.5–0.8 per 1,000,000 individuals (Afro-Americans more likely) in a 10-year period, before the COVID-19 pandemic [9,59,60]. More recently, a significantly higher rate (one case per 100,000 people) was reported among recipients of the ChAdOx1 nCoV-19 vaccine (especially women) in an editorial by Ropper and Klein [60] and by the EMA (one per 250,000 people) [61].

More dramatic rates have been described by Pottegård et al. from Danish and Norwegian registries, with one case in 40,160 (24.9 per 1,000,000) [62]. In this regard, however, discrepancies in delivering vaccination among countries should also be considered, especially at early vaccination campaign, as vaccines were not easily available [60,61].

From the findings of the present study, it appears that mRNA vaccines and the recent Vaxzevria (just one patient in our data set) are safer than the native ChAdOx1 nCoV-19 vaccine, with respect to the occurrence of CVST.

Of interest, similar events also occurred in COVID-19 patients [63]. In a retrospective cohort study, CVST was found in 40–45 patients per 1,000,000 infected [9]. The more serious the infection, there was more likelihood of vascular thrombotic complications as reported by Piazza et al. [64], with 35% incidence in hospitalized vs. 2.6% non-hospitalized patients in the intensive care unit. Therefore, some pathophysiological similarities can be postulated to cause CVST in both infected and vaccinated patients [36,58,60,61,62,63,64].

Historically, prognosis in CVST is strictly related to its specific clinical setting, quick diagnosis, and treatment protocols. The mortality rate has been reported to vary from 5 to 15% and more than two-thirds of patients make a full recovery [9,57,59,60,65,66].

Unfortunately, mortality rates in our data set were very high among complicated patients, in whom brain injuries, whether associated with severe thrombocytopenia, were strong prognosticators. Anti-thrombotic treatment was more effective in uncomplicated patients.

### 4.1. Pathophysiological Mechanism(s) of Thrombosis

At the time of this review, CVST is considered a VITT framework. However, in the study of Pavord et al. from the UK [38], anti-PF4 antibodies were present in 198 of 220 patients (90%) with definite/probable VITT, and 81% of those who died. Only 17 specialist laboratories in the UK were able to perform the HIT-ELISA method for testing anti-PF4 antibodies. In the studies included in our review, such laboratories were hardly accessible at least until May 2021, thus explaining anti-PF4-positivity only in half of patients. Of clinical interest, however, a recent study by Terpos et al. demonstrated that antibodies against PF4 were found in two-thirds of ChAdOx1 nCoV-19 recipients irrespective of clinical manifestation of thrombosis, to some extent related to COVID-19 virus-neutralizing circulating antibodies [67]. These data, if confirmed, may raise questions regarding the genetic immunity problem prior to the vaccine. Based on inherent studies, position papers, and current findings [3,52,57,58,59,60,61,65], the main predisposing factors for CVST after COVID-19 vaccination, primarily adenoviral vector, are summarized in Table 3.

Of note, most CVST patients in this review had no typical prothrombotic risk factors, and this contributed to the media reports on such unexpected outcomes.

As aforementioned, blood clots can be triggered by abnormal immunogenicity of PF4–polyanion complexes. PF4 is a protein present in the a-granules of platelets that can quickly bind either exogenous or endogenous polyanions such as heparin, bacterial or virus proteins, or glycosaminoglycans of the vascular cells. The PF4/polyanion pair act as autoantigen-inducing antibodies of the IgG class. On the other hand, PF4–polyanion–IgG complexes can also be detected in 3–5% of healthy subjects, with a risk of platelet aggregation via FcRIIa/CD32 receptors on their surface, which could be a common pathway for VITT, HIT, or DIC syndromes [4,5,7,8,16,57,58]. Similarly, spike proteins during COVID-19 infection have also been demonstrated to cause PF4 degranulation, platelet aggregation, and thrombosis [4,64,65].

Although CVST was detected mainly among women younger than 60 years, 169 men also showed the same complication. Coagulative disorders in pregnant and puerperal women were found to cause CVST as a manifestation of DIC/HIT-like syndromes. Hormonal alterations and thromboplastin-like material from the amniotic fluid have been suggested as potential triggers of vascular thrombosis [7,8,9,57,58]. However, questions regarding male patients remain open. Antiphospholipid antibody syndrome, factor V Leiden disorders, protein S and/or protein C deficiency, prothrombin gene mutation, and hyper-homocysteinemia may be considered in both women and men, although these features have been found in approximately 30% of CVST patients in previous studies [9,57,68]. Oral contraceptives, drugs, cancer, chemotherapy, leukemia, polycythemia, neurological diseases (cranial traumas, meningiomas, neurosurgical procedures, bacterial meningitis, hydrocephalies) can also emblematize hazardous conditions [59,60,68].

### 4.2. Cerebral Venous Compartment

The whys and wherefores of CVST that may lead to cerebral tissue injury have been described previously [10,53,56,57,59,60]. In the first clotting phase, occlusion is incomplete, the venular pressure gradually increases, capillary exchanges start failing, cerebrospinal fluid absorption relents, and edema with tissue ischemia occurs. As thrombi become occlusive or disseminated, the arteriolar and venular wall, and capillaries as well, become highly friable and bleeding. The reasons why thrombosis often occurs in the cerebral venous vessels have been debated; this issue needs to be studied. Randomness is not enough to explain such unusual phenomena [57,61,62].

We have learned from Virchow’s studies that at least three components are needed for red clot formation within the venous system [69]. Given that PF4–immune complexes are important clues for the VITT syndrome [5,8,13,55,57], other cofactors such as slow blood flow velocity and endothelial disease should also be encountered for thrombogenicity [69,70,71]. We do not know whether blood flow slowing may occur within cerebral veins in the absence of anatomical anomalies. However, we are aware of the fact that even apparently healthy people (women first) suffer from inherited venous hypotonia [72] that may result in subclinical phlebostasis. In our patients with blood clot site description, thrombosis chiefly occurred within transverse, sigmoid, and superior sagittal sinuses, confirming previous studies in both adults and children [59,64]. These findings would suggest their flat, transversal, pathway might be implicated in thrombogenesis, together with other predisposing features. Studies have demonstrated that extracranial compression or anomaly of the internal jugular vein concurs to red cell sludging and thrombosis in intracranial tributary veins [59,73,74]. Jugular vein pressure, as an expression of the right atrial pressure, was recently reported as being predictive of central venous congestion [75].

### 4.3. Allergic Reactions

Anaphylaxis represents another important issue to consider. The immune system produces neutralizing antibodies to membrane proteins of pathogens, such as adenoviral vectors, spike proteins, and mRNA-stimulated recipient cells. Lymphocytes and neutrophil extracellular traps (NETs) are inflammatory modulators and can activate the coagulation cascade through factor VII and other tissue factors, possibly causing VITT in both infected and vaccinated individuals. Furthermore, genetic polymorphism, high endothelial CD32 receptor’ density, and/or different vascular affinity for such immune complexes may be further cofactors of the immunomodulated vasculitis and thrombosis [2,4,36,76,77,78], as confirmed by immuno-histochemical post-mortem examinations showing the coexistence of multisite vessel thrombosis and cell-mediated endothelial inflammation [24,40,48].

Thrombosis may be the consequence of allergic reactions to vaccine excipients, often occurring days after the shot. These are usually antibody independent, cell mediated stemming from T-cell activation, as well as monocyte and macrophage responses to the sensitizer [2,77,78]. Vaccines containing thimerosal, aluminum, polyethylene glycol, polysorbate 80, and other molecules can determine either immediate or delayed hypersensitivity. Platelet’s surface shows a high affinity for IgE via FcRI receptors and low-affinity IgE FcRII/CD23 receptors, and the subsequent cytokine storm closely resembles the pathophysiology of Kounis syndrome [2,4]. Both ChAdOx1 nCov-19 and Ad26.COV2 vaccines contain polysorbate 80, a synthetic nonionic surfactant additive that may convey severe allergic reactions and vascular thrombosis. Of clinical concern, polysorbate 80 also breaks the blood–brain barrier, enhances membrane permeability, and facilitates the migration of drugs or other substances from the blood compartment into the brain [2,78,79].

### 4.4. Other Hypotheses

There are many unsolved issues on COVID-19 disease and related inflammatory reaction injuries determining clinical phenotype among infected individuals. The role of neutralizing antibodies, B- and T-cells, white cells, platelets, interleukins, and interferon represent a myriad of features for preventing organ disease infection. Immunopathogenesis of both infection- and vaccine-related adverse effects is continuously under investigation. Acute respiratory distress syndrome may be caused by heterogeneous events, including non-infectious agents. It is a recognized notion that inflammation can be triggered even by small substances derived from injured, post-traumatic and/or post-vaccine, targeted cells (either infected or non-infected). These pathogen-like substances can lead to unusual clinical pictures such as the multisystem inflammatory syndrome in children or multisite thrombogenic syndrome in adults [79,80].

To explain all clinical aspects of immunological diseases, new concepts of immunopathogenesis are now accepted, in which not solely pathogens themselves but also infection-associated immune-mediated responses and/or vaccine-induced complications (vascular thrombosis) may have a ‘limited’ ability to cause host cell injury. It is now known that the host immunity reacts to not only smaller substances from the infectious agent, including toxins and pathogen-associated molecular patterns (PAMPs), but also to pro-inflammatory proteins and peptides produced by the injured (non-infected) host cells, known as damage-associated molecular patterns (DAMPs). It has been postulated that the main function of the host immune/repair system at the molecular level is to control the release of toxic substances by a better identification of inflammatory proteins and peptides, based on their size and biochemical characteristics (the *protein-homeostasis-system hypothesis*) [80,81,82,83].

## 5. Study Limitations

There are some limitations in the present narrative review. We included studies afterthorough review, some studies could have been missed. A few studies we considered as original descriptions were also included in subsequent reviews from the same region or study group, and it was difficult to extrapolate these duplicates from inclusive and larger case series.

This research was not for a pharmaco-vigilance purpose, so the event rates were just hypothesized based on previous studies. In fact, stratification of rare events among larger populations can be inaccurate unless all events had been noticed by the Health Authority. The best way to evaluate the CVST risk should be by performing a pair-matching analysis on vaccinated vs. unvaccinated people in a 1:1 ratio, according to their demographic and clinical characteristics. As mentioned, in early 2021, there was slight clinical awareness on post-vaccine syndromes, so some cases could have been missed. At present, this represents a hypothesis-generating study, and further investigation on chemical and pharmacological properties, production, and delivery of vaccines is encouraged.

## 6. Conclusions

Vaccines remain the best way to fight the current COVID-19 pandemic. The present study likely confirmed the rare, often dramatic, occurrence of CVST following (adenovirus vector) vaccine shots. The incidence of thrombus formations was 2.25-fold higher in women of childbearing age than in men, and systemic multivessel disease was detected in >90% of cases. Transverse and sigmoid venous sinuses were preferred intracerebral locations for thrombosis, and poor outcomes were reported in patients with brain injury, such as intracranial bleeding, whether in association with thrombocytopenia. Although thrombogenic mechanisms are still under discussion within the scientific community, currently available histopathological findings indicate an underlying prothrombotic immune micro- and macro-vasculitis.

## Figures and Tables

**Table 2:** Summarized criteria for VITT syndrome from our CVST patient population (32/41 studies).

Criteria *	Proportion of Patients
COVID vaccine 4–42 days prior to symptom onset	98%
Any venous or arterial thrombosis	100%
Thrombocytopenia	61%
Positive PF4-polyanion-Ig by ELISA	47%
D-dimer > 4 times upper limit of normal	80%

* Criteria for vaccine-induce thrombotic thrombocytopenia (VITT), according to American Society of Hematology Guidelines (https://www.hematology.org/covid-19/vaccine-induced-immune-thrombotic-thrombocytopenia, accessed on 30 November 2021); Greinacher et al. [5]; Shultz et al. [41]; Sharifian-Dorche et al. [53]; Rizk et al. [52]; and Lee et al. [54].

**Table 3:** Predisposing conditions for CVST to consider in people undergoing (adenoviral vector) vaccination (based on former studies).

Autoimmune/inflammatory disorders
African Americans
Cancer
Endocranial hypertension
Genetic or acquired pro-thrombotic conditions
History of allergy to any of the vaccine components
History of chronic headache of unknown origin
History of peripheral phlebothrombosis
History of sinusitis and meningitis
Immunosuppressive treatment
Previous (minor or major) stroke
Thrombocytopenia/thrombocytosis
